# PFDN2 promotes cell cycle progression via the hnRNPD-MYBL2 axis in gastric cancer

**DOI:** 10.3389/fonc.2023.1164070

**Published:** 2023-07-18

**Authors:** Qiuming He, Zheyu Ding, Tingna Chen, Haitao Wu, Jialing Song, Zhenxian Xiang, Chaogang Yang, Shuyi Wang, Bin Xiong

**Affiliations:** ^1^Department of Gastrointestinal Surgery & Department of Gastric and Colorectal Surgical Oncology, Zhongnan Hospital of Wuhan University, Wuhan, China; ^2^Department of Gastroenterology, The Central Hospital of Wuhan, Tongji Medical College, Huazhong University of Science and Technology, Wuhan, China; ^3^Hubei Key Laboratory of Tumor Biological Behaviors, Wuhan, China; ^4^Department of Thyroid and Breast Surgery, Maternal and Child Health Hospital of Hubei Province, Tongji Medical College, Huazhong University of Science and Technology, Wuhan, China

**Keywords:** PFDN2, Cell Cycle, MYBL2, gastric cancer, HNRNPD

## Abstract

Gastric cancer (GC) is a major health burden worldwide, but our understanding of GC is limited, and the prognosis is poor. Novel therapeutic strategies and biomarkers are urgently needed to improve GC patient outcomes. Previously, we identified PFDN2 as a novel key gene in gastric cancer based on its differential expression between cancer and normal tissues. However, the role and underlying mechanisms of PFDN2 in GC remain elusive. In this article, we demonstrated that PFDN2 is highly expressed in GC and that upregulation of PFDN2 is associated with the progression of GC. We further found that PFDN2 could promote cell cycle progression by promoting MYBL2 expression. Mechanistically, we demonstrated that PFDN2 could upregulate MYBL2 expression by facilitating the nuclear translocation of hnRNPD, and thus promoting MYBL2 transcriptional program. In conclusion, we found that PFDN2 promotes cell cycle progression via the hnRNPD-MYBL2 axis and may serve as a potential biomarker and therapeutic target for GC.

## Introduction

Gastric cancer (GC) is the fourth most common cancer and has a high mortality rate around the world (1).Despite substantial therapeutic advancements, including chemotherapy, radiation therapy, targeted therapies, and epigenetic agents, the prognosis of GC patients remains poor (2). Consequently, there is an urgent need for a better understanding of the mechanisms underlying the occurrence and development of GC, which may be helpful for GC diagnosis and treatment.

With the rapid development of sequencing technology and bioinformatics, we were able to identify many key genes involved in the development and progression of GC, which may be effective as tumor biomarkers or therapeutic targets. Previously, we identified PFDN2 as a novel key gene in gastric cancer based on its differential expression between cancer and normal tissues (3). The PFDN family consists of six members (PFDN1-6), which have been reported to be associated with the development and progression of multiple types of cancer (4, 5). For instance, PFDN1 can directly repress the transcription of cyclin A and thus promote lung cancer development (6); outlier PFDN2 expression can be detected in urine, which could serve as a biomarker for bladder cancer (7); PFDN5 could modulate c-MYC transcriptional regulatory activity (8). However, the role of PFDN2 in cancer initiation and progression remains unknown, especially in GC.

MYBL2, a member of the myb gene family, plays an important role in regulating the cell cycle through interaction with other cell cycle transcription regulators and activating target gene transcription (9). For example, MYBL2 promotes cell cycle progression through reciprocal feed-forward transactivation of E2F2 in colorectal cancer (10). In addition, many G2/M genes were reported to be regulated by MYBL2 in breast cancer (11). Nevertheless, few studies have determined the molecular mechanism of MYBL2 upregulation in GC.

In this article, we demonstrated that PFDN2 was highly expressed in GC and that upregulation of PFDN2 was associated with the progression of GC. We further found that PFDN2 could promote cell cycle progression by promoting MYBL2 expression. Mechanistically, we demonstrated that PFDN2 could upregulate MYBL2 expression by facilitating the nuclear translocation of hnRNPD, and thus promoting MYBL2 transcriptional program. Our study revealed the role of PFDN2 in GC cell cycle progression and may provide a novel tumor marker and a potential target for GC.

## Materials and methods

### Patients

GC and adjacent noncancerous tissue samples were obtained from 50 patients with gastric cancer at the Zhongnan Hospital of Wuhan University. None of the patients underwent any preoperative radiotherapy or chemotherapy. The study was approved by the Ethics Committee of Zhongnan Hospital, Wuhan University. Informed consent was obtained from all patients.

### Cell culture and transfection

The normal gastric epithelium cell line GES-1 and GC cell lines (AGS MGC-803 HGC-27 MKN-45 SGC-7901) were purchased from the Chinese Academy of

Sciences. All cell lines were authenticated prior to their use and tested for mycoplasma contamination every 3 months. All GC cells were cultured at 37 °C with a 5% CO_2_ atmosphere in 1640 (GIBCO, USA) supplemented with 10% FBS (GIBCO, USA). siRNA and negative control siRNA transfections were performed using Lipofectamine 2000 (Thermo Fisher Scientific, Waltham, USA). The target sequences were as follows: si-PFDN2-1: GCUUCAACCGCCUUCGGCATT; si-PFDN2-2: CCGCUUUGGAGAACAACAATT; si-PFDN2-3: GCUGGAGUGUUGGUCUCCUTT; si-MYBL2: CAGACAAUGCUGUGAAGAATT; si-hnRNPD: AGACUGCACUCUUGAAGUUATT. The lentiviral construct containing shRNA against PFDN2 was purchased from Gene Pharma (Shanghai, China). Lentiviral transfection was performed according to the provided protocol.

### Quantitative real-time PCR (qRT−PCR)

Total RNA from cells and patients was isolated using TRIzol (Invitrogen, USA). RNA concentrations were measured using a NanoDrop 2000 (Thermo Fisher Scientific). The RNA (1 µg) was reverse-transcribed using PrimeScript RT reagent Kit (Vazyme, China). qRT−PCR was performed using SYBR Green master mix (Vazyme, China). The primer sequences were as follows: PFDN2-fw: 5′-ATGAGCACAGCCTAGTGATCG-3′; PFDN2-rev: 5′-ACTCCTCCAACCATGCGGTA-3′.

### Western blot assay

Samples for protein analysis were extracted on ice using RIPA lysis buffer. After centrifugation, the supernatants were quantified using a bicinchoninic acid (BCA) kit. Then, the samples were boiled in SDS sample buffer and electrophoresed by 10% SDS–PAGE. After being transblotted onto PVDF membranes, blots were incubated with primary antibodies overnight at 4 °C and then incubated with secondary antibodies for 1 h at room temperature. The antibodies used were as follows: anti-PFDN2 (Novus, NBP3-00279, 1:1000); anti-MYBL2 (Proteintech, 18896-1-AP, 1:300); anti-hnRNPD (Abcam, ab259895, 1:1000); anti-P21 (Proteintech, 2947S, 1:1000); anti-CDK4 (Proteintech, 12790S, 1:1000); anti-CDK6 (Proteintech, 13331S, 1:1000); anti-cyclin D1 (Proteintech, 55506S, 1:1000); and anti-GAPDH (Proteintech, 60004-1-Ig, 1:5000).

### EdU assay

EdU analysis was processed using the BeyoClick™ EdU Cell Proliferation Kit (Beyotime, C0071L, China) in accordance with the manufacturer’s instructions. Images were taken using a fluorescence microscope.

### Wound healing assay

GC cells were seeded in 6-well plates and grown until ~80% confluence. A sterile pipette tip was used to make the scratch line. The migration rate was calculated using ImageJ software.

### Transwell migration and invasion assay

Transwell chambers (8 μm pore size; Corning, USA) were used for the migration assay. Cell invasion assays were performed with Matrigel-coated Transwell chambers. MKN45 cells and HGC27 cells were seeded in the upper chamber and incubated for 48 h. Then, the migrated and invaded cells were fixed in 4% paraformaldehyde and stained with 0.5% crystal violet. Five random fields from each well were counted under a microscope (magnification, ×200).

### RNA-seq

Total RNA from the NC and PFDN2-KD groups of HGC27 cells was extracted using TRIzol reagent (Invitrogen). Quality control, library construction, RNA sequencing, and bioinformatic analysis were performed at BGI (Beijing Genomics Institute). The GSEA for differentially expressed genes was performed on the Dr. Tom network platform of BGI (http://report.bgi.com).

### Coimmunoprecipitation (CoIP) and MS/MS analysis

The CoIP assay was performed using an IP/CoIP Kit (#abs955, Shanghai, China). Briefly, GC cells were lysed on ice. After centrifugation, the supernatants were incubated with anti‐PFDN2 (Novus, NBP3-00279) or normal rabbit IgG (CST, #3900, USA) with rotation at 4°C overnight. The next day, Protein A/G was added to the supernatants with rotation at 4°C for 3 h. Finally, the supernatants were analyzed using MS/MS and WB.

### Subcutaneous tumorigenesis experiment

BALB/c nude mice (female, 4‐week‐old) were purchased from Hubei Research Center of Laboratory Animals (Wuhan, China). PFDN2-KD/NC HGC27 cells (5 × 10^6^) were injected subcutaneously into the right flank region of nude mice. After 5 weeks, the mice were anesthetized and sacrificed. The tumor tissues were collected for tumor weight and other analyses. Animal experiments were approved by the Ethics Committee of Zhongnan Hospital, Wuhan University (ZN2022128).

### Statistical analysis

Statistical analyses were performed using SPSS 17.0 (SPSS Inc., USA). Student’s t test was used to analyze the data. Kaplan−Meier analysis was used to analyze overall survival and progression-free survival. The data are presented as the mean ± SD in the figures. P < 0.05 was considered statistically significant.

## Results

### Upregulation of PFDN2 is associated with the progression of gastric cancer

**Figure 1:**
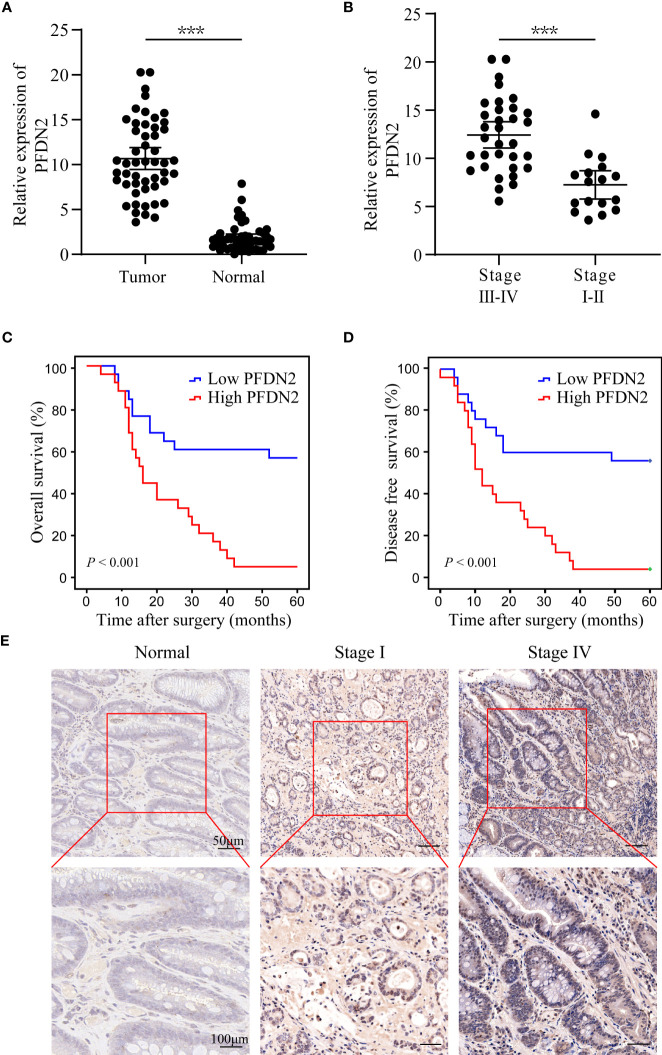
Upregulation of PFDN2 is associated with the progression of gastric cancer. **(A)** qRT–PCR analysis of PFDN2 expression levels in GC and normal tissues. **(B)** PFDN2 level was significantly greater in GC with higher grade. **(C, D)** The associations of PFDN2 expression with five-year OS and five-year DFS. **(E)** IHC staining analysis of PFDN2 in low stage GC, high stage GC and normal tissues. ***p < 0.001.

We previously identified PFDN2 as a novel key gene in gastric cancer (GC) based on its differential expression between cancer and normal tissues (3). The expression of PFDN2 was analyzed in 50 pairs of GC specimens and matched distal normal tissues by quantitative real-time PCR. We found that PFDN2 was significantly overexpressed in GC, especially in tumors with higher stages (**Figures 1A, B**). Furthermore, Kaplan‐Meier survival curves showed significantly lower overall survival in GC patients with high PFDN2 expression (**Figures 1C, D**). Finally, IHC staining further confirmed that PFDN2 is highly expressed in GC, especially in tumors with higher stages (**Figure 1E**). Collectively, these data indicate that PFDN2 is highly expressed in GC and its upregulation is correlated with higher clinical stage and worse prognosis.

### PFDN2 enhances GC cell proliferation, migration, and invasion

**Figure 2:**
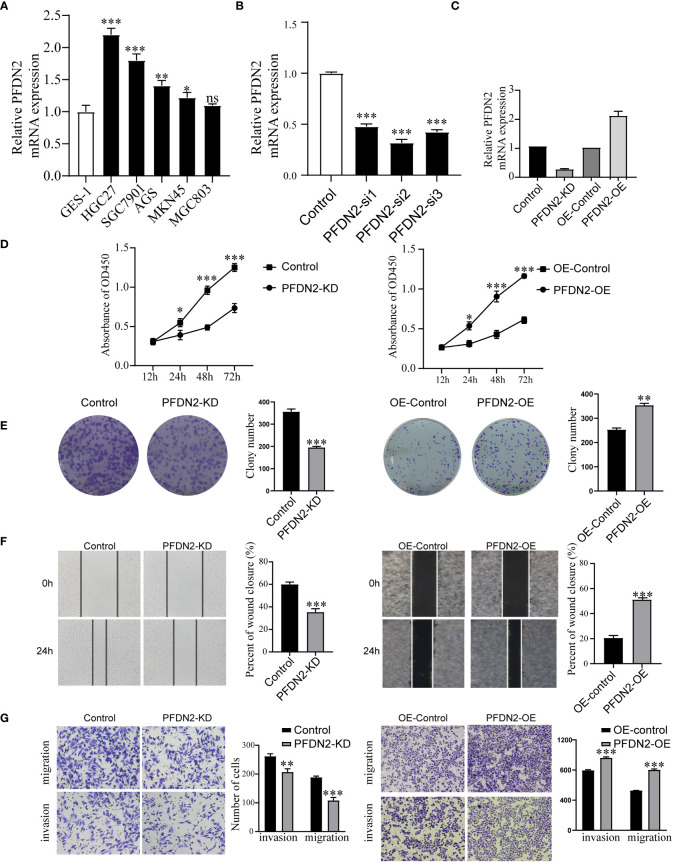
PFDN2 enhances GC cell proliferation, migration, and invasion. **(A)** qRT-PCR analysis of PFDN2 expression level in GES-1 and GC cells lines. **(B)** Three siRNAs was used in HGC27 to knockdown PFDN2. **(C)** Construction of the PFDN2-KD/OE stable cell lines. **(D)** CCK8 assay was performed in GC cell lines following PFDN2 knockdown or overexpression. **(E)** Colony formation assays were performed after PFDN2 knockdown or overexpression in HGC27 or MKN45, respectively. **(F)** Wound healing assay was performed in GC cell lines following PFDN2 knockdown or overexpression. **(G)** Migration and invasion assays of GC cell lines after PFDN2 knockdown or overexpression. *p < 0.05; **p < 0.01; ***p < 0.001; ns, no significance

Next, we examined PFDN2 expression in GC cell lines and found that PFDN2 was highly expressed in most cancer cells, especially HGC27 cells (**Figure 2A**). To investigate the biological function of PFDN2, a pool of three siRNAs was used in HGC27 cells to knockdown PFDN2 (**Figure 2B**). Among the three siRNAs, Si-2 had the best knockdown effect and was used to establish PFDN2 knockdown (KD) stable cell lines by lentiviral transduction. Likewise, PFDN2 overexpression (OE) was performed in MKN45 cells using a lentivirus system (**Figure 2C**). Next, CCK-8 assays (**Figure 2D**), and colony formation assays (**Figure 2E**) were conducted to investigate the effect of PFDN2 on cell proliferation. The results showed that PFDN2 overexpression significantly promoted MHC803 cell growth, while PFDN2 knockdown inhibited HGC27 cell proliferation. Furthermore, the results of the scratch and Transwell assays demonstrated that PFDN2 overexpression promoted cell migration and invasion (**Figure 2F, G**). Conversely, PFDN2 knockdown inhibited cell migration and invasion.

### PFDN2 promotes cell cycle progression in GC cells

**Figure 3:**
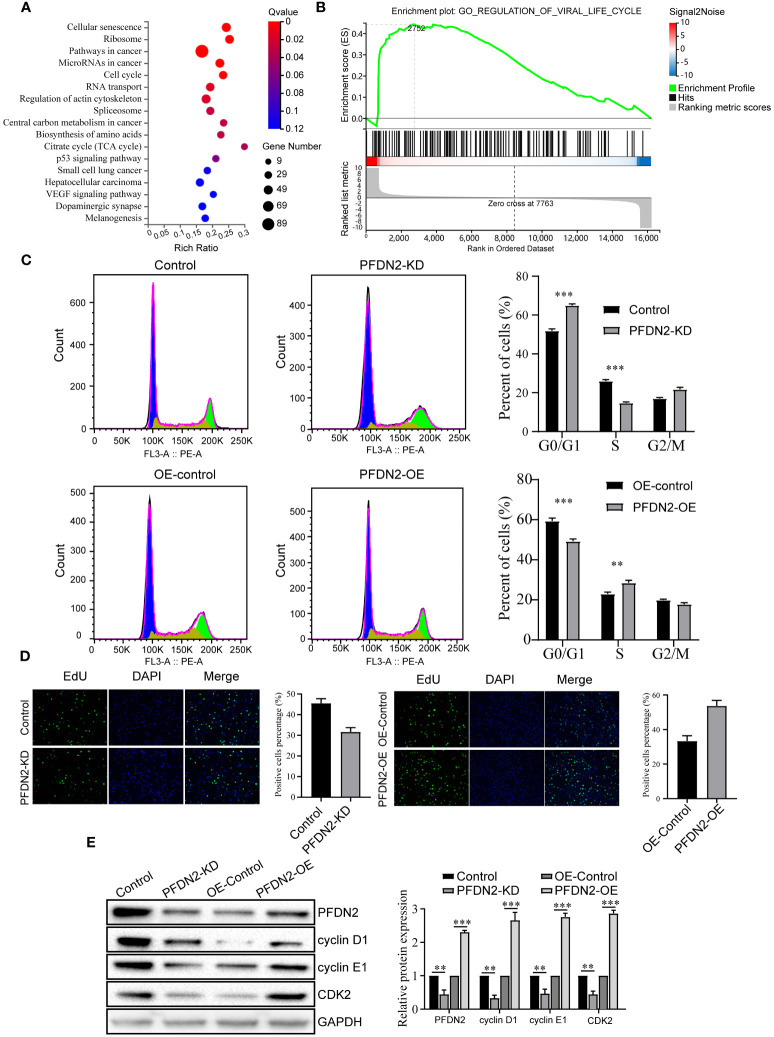
PFDN2 promotes cell cycle progression in GC cells. **(A)** Bubble chart showing Gene Ontology (GO) enrichment analysis of the RNA-sequencing (RNA-seq) data from PFDN2-KD and HGC27-NC cells. **(B)** GSEA of the RNA-sequencing (RNA-seq) data from PFDN2-KD and HGC27-NC cells. **(C)** Flow cytometry was used to analyze the cell cycle in HGC27-NC and PFDN2-KD cell lines and in MKN45-NC and PFDN2-OE cell lines. **(D)** Cell proliferation abilities were determined by EdU staining in HGC27-NC and PFDN2-KD cell lines and in MKN45-NC and PFDN2-OE cell lines. **(E)** WB analysis of PFDN2, cyclin D1, cyclin E1 and CDK2 in HGC27-NC and PFDN2-KD cell lines and in MKN45-NC and PFDN2-OE cell lines. The protein levels were quantified with ImageJ. **p < 0.01; ***p < 0.001.

To explore the mechanism by which PFDN2 promotes GC cell proliferation, migration, and invasion, we performed RNA transcriptome sequencing of control or PFDN2-KD HGC27 cells, and differentially expressed genes (DEGs) were subjected to GSEA for pathway enrichment analysis. The results revealed that these DEGs were significantly enriched in the “cell cycle” (**Figures 3A, B**). Thus, we next investigated the effects of PFDN2 on the cell cycle in GC cells through flow cytometry and EdU assays. As shown in **Figure 3C**, PFDN2 knockdown increased the percentage of cells in the G1 phase of the cell cycle. In contrast, PFDN2 overexpression decreased the percentage of cells in the G1 phase. Conversely, the EdU assay revealed that PFDN2 overexpression enhanced DNA biosynthesis and that PFDN2 knockdown reduced DNA biosynthesis (**Figure 3D**). Furthermore, we found that PFDN2 overexpression increased the expression of G1/S phase transition-associated proteins (cyclin D1, cyclin E1 and CDK2), while PFDN2 knockdown reduced the expression of these proteins (**Figure 3E**). Taken together, these results demonstrate that PFDN2 promotes cell cycle progression in GC cells.

### PFDN2 promotes cell cycle progression by regulating MYBL2 expression

**Figure 4:**
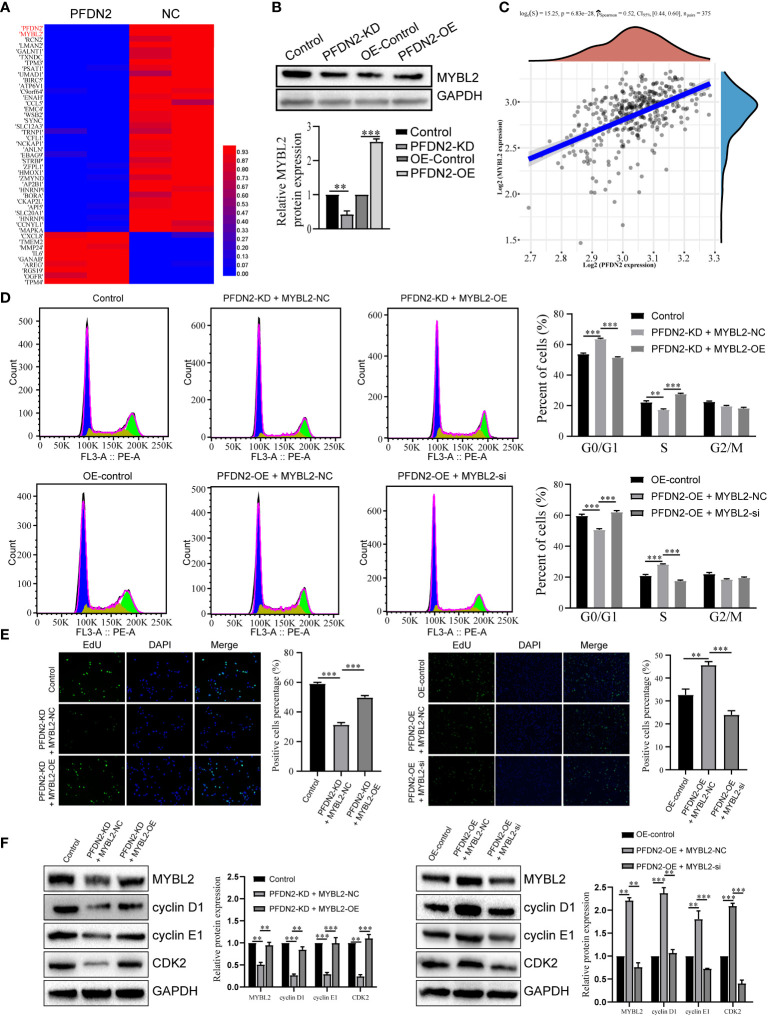
PFDN2 promotes cell cycle progression through regulating MYBL2 expression. **(A)** Heatmap presenting significantly differentially expressed transcripts between HGC27-NC and PFDN2-KD cell lines. **(B)** WB analysis of MYBL2 in HGC27-NC and PFDN2-KD cell lines. **(C)** The correlation of PFDN2 expression with MYBL2 expressions in GC tissues from TCGA. **(D)** Flow cytometry was used to analyze the cell cycle in HGC27-NC and PFDN2-KD cell lines treated with MYBL2 OE or NC and in MKN45-NC and PFDN2-OE cell lines treated with MYBL2 KD or NC. **(E)** Cell proliferation abilities were determined by EdU staining in HGC27-NC and PFDN2-KD cell lines treated with MYBL2 OE or NC and in MKN45-NC and PFDN2-OE cell lines treated with MYBL2 KD or NC. **(F)** WB analysis of PFDN2, cyclin D1, cyclin E1 and CDK2 in HGC27-NC and PFDN2-KD cell lines treated with MYBL2 OE or NC and in MKN45-NC and PFDN2-OE cell lines treated with MYBL2 KD or NC. **p < 0.01; ***p < 0.001.

We next explored the specific mechanism by which PFDN2 promotes GC cell cycle progression. By RNA sequencing of control or PFDN2-KD HGC27 cells, we identified 22 downregulated genes (fold change ≥2) and 53 upregulated genes (fold change ≤0.5) (**Figure 4A**; **Supplementary Table 1**). Among them, MYBL2 was the most downregulated gene, except PFDN2 itself, caused by PFDN2 knockdown. WB analysis validated the sequencing results (**Figure 4B**). Furthermore, correlation analysis from the TCGA database showed that PFDN2 was positively correlated with MYBL2 in GC (**Figure 4C**). MYBL2 is a core regulator of cell cycle progression in the development of human cancers (9). Thus, we next explored whether PFDN2 promotes cell cycle progression by regulating MYBL2 expression. First, siRNA was used to knockdown MYBL2, and a MYBL2 expression plasmid was used to overexpress MYBL2 in GC cells. We explored whether MYBL2 could rescue the effect of PFDN2 on cell cycle progression. The results showed that MYBL2 knockdown could rescue PFDN2-OE-regulated cell cycle progression and that MYBL2 overexpression could also rescue PFDN2-KD-regulated cell cycle progression (**Figures 4D–F**). The above results demonstrated that MYBL2 is essential for PFDN2-mediated cell cycle progression.

### PFDN2 regulates MYBL2 expression by binding with hnRNPD

**Figure 5:**
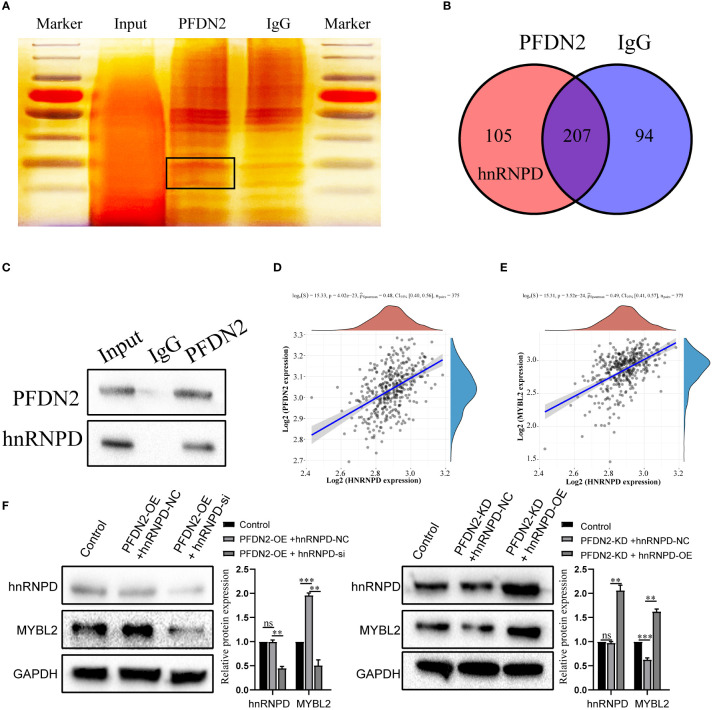
PFDN2 regulates MYBL2 expression through binding with hnRNPD. **(A)** Silver staining of PFDN2-interacting proteins by co-IP expriments. Black boxes indicate specific bands. **(B)** Identification of PFDN2 binding partners by mass spectrometry. **(C)** CoIP of PFDN2 or hnRNPD detected by western blotting. **(D)** The correlation of PFDN2 expression with hnRNPD expressions in GC tissues from TCGA. **(E)** The correlation of MYBL2 expression with hnRNPD expressions in GC tissues from TCGA. **(F)** WB analysis of MYBL2 and hnRNPD in HGC27-NC and PFDN2-KD cell lines treated with hnRNPD OE or NC and in MKN45-NC and PFDN2-OE cell lines treated with hnRNPD KD or NC. **p < 0.01; ***p < 0.001; ns, no significance

Next, we further investigated the molecular mechanisms by which PFDN2 upregulated MYBL2. The PFDN family of proteins can interact with different proteins and play various roles in tumor progression (12). Thus, we used coimmunoprecipitation (co-IP) and mass spectrometry (MS) to identify potential PFDN2 binding proteins. 105 proteins specifically bound to PFDN2 were identified (**Figures 5A, B**; **Supplementary Table 2**). Among them, hnRNPD, which immediately caught our attention, was significantly enriched in the anti-PFDN2 group. HnRNPD is part of the hnRNP family and has been reported to function as an oncogene in a variety of cancers (13, 14). WB for coprecipitation was used to further demonstrate that PFDN2 binds to MYBL2 (**Figure 5C**). In addition, hnRNPD expression was significantly associated with PFDN2 and MYBL2 expression in TCGA datasets (**Figures 5D, E**). Finally, we altered MYBL2 expression to investigate whether PFDN2 regulated MYBL2 expression by binding with hnRNPD. HnRNPD overexpression reversed the downregulation of MYBL2 upon PFDN2 knockdown, and hnRNPD knockdown reversed the upregulation of MYBL2 upon PFDN2 overexpression (**Figure 5F**). In summary, our results demonstrated that PFDN2 regulates MYBL2 expression by binding with hnRNPD.

### PFDN2 promotes MYBL2 transcriptional program by facilitating the nuclear translocation of hnRNPD

**Figure 6:**
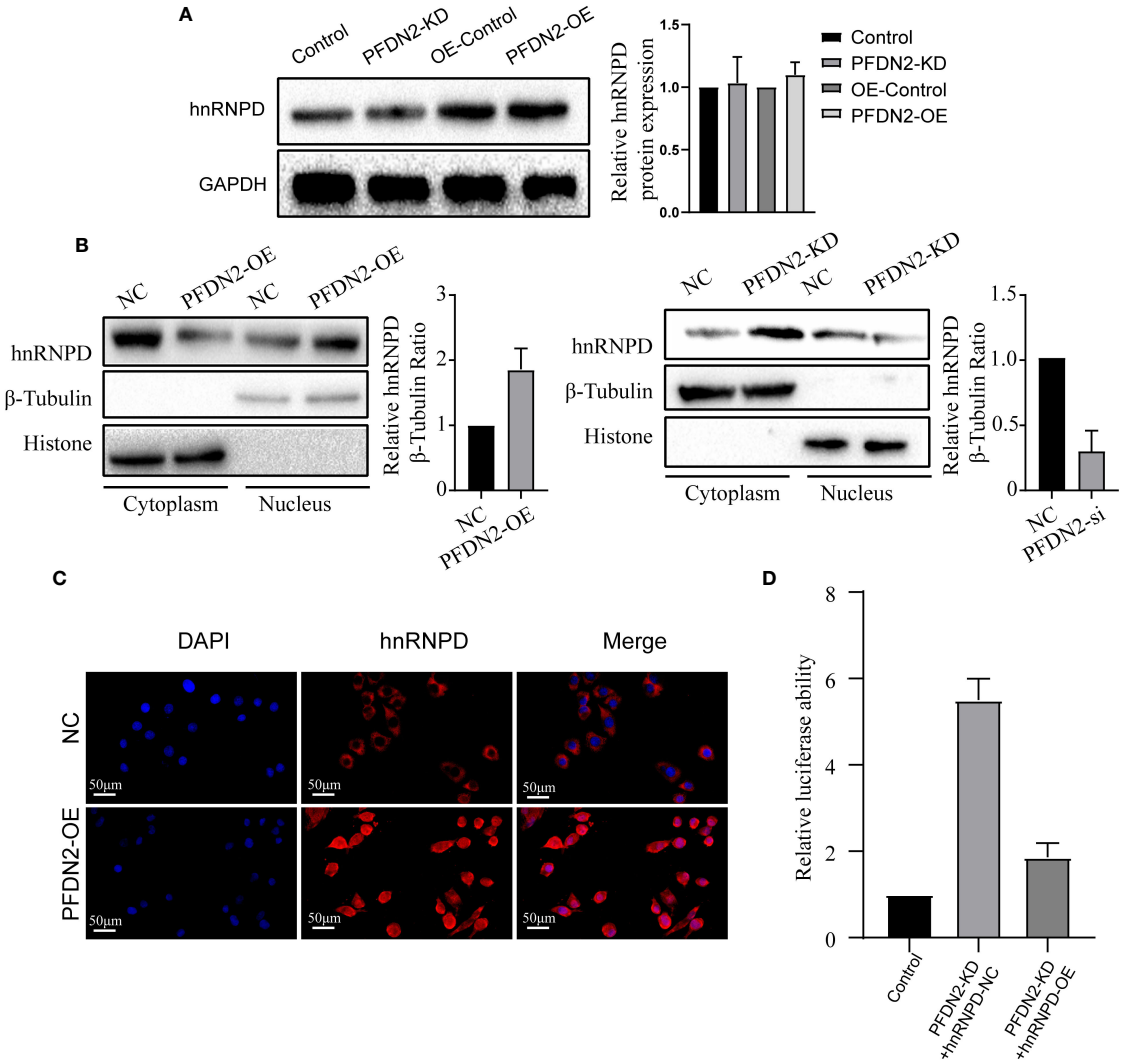
PFDN2 promotes MYBL2 transcriptional program by facilitating the nuclear translocation of hnRNPD. **(A)** PFDN2 could not regulate hnRNPD expression. **(B)** WB showing hnRNPD levels in the nucleus and cytoplasm of GC cells. **(C)** Immunofluorescence staining for hnRNPD in MKN45-NC and PFDN2-OE cell lines. **(D)** Luciferase assay showing the transcriptional activity of MYBL2 in GC cells.

Our above results demonstrated that PFDN2 regulated MYBL2 expression through binding with hnRNPD, so we next sought to explore the underlying mechanisms. First, we investigated if PFDN2 could regulate hnRNPD expression. However, our results shown that neither PFDN2 knockdown or overexpression altered hnRNPD expression (**Figure 6A**). Previously, it has been shown that nuclear translocation of hnRNPs could regulates downstream gene transcription (15, 16). So, we next explore whether PFDN2 could facilitate the nuclear translocation of hnRNPD and thus upregulated MYBL2. Interestingly, our experimental findings shown that PFDN2 significantly enhanced nuclear hnRNPD colocalization (**Figure 6B**). These observations were further validated by immunofluorescence (**Figure 6C**)

We next wanted to address the detailed mechanism by which PFDN2 upregulates MYBL2 expression. Previous studies indicate that hnRNP family have the inherent capacity to promote transcription (6). Therefore, we examined whether PFDN2 regulate MYBL2 transcription through hnRNPD. Significantly, dual-reporter luciferase assays showed that overexpression of PFDN2 in MKN45 cells stimulated promoter activity of MYBL2, and hnRNPD knockdown reversed this effect (**Figure 6D**). These findings indicate that PFDN2 promotes MYBL2 transcriptional program by facilitating the nuclear translocation of hnRNPD.

### PFDN2 promotes GC tumorigenesis *in vivo*

**Figure 7:**
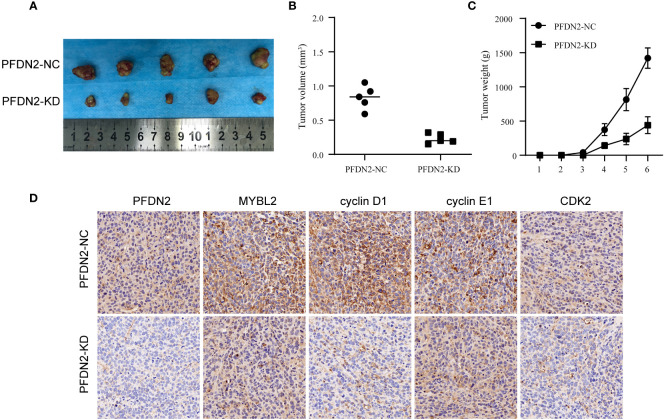
PFDN2 promotes GC tumorigenesis *in vivo*. **(A)** The morphological characteristics of subcutaneous tumor xenografts in HGC27-NC and PFDN2-KD groups. **(B)** Subcutaneous tumor volume in HGC27-NC and PFDN2-KD groups. **(C)** Subcutaneous tumor weight in HGC27-NC and PFDN2-KD groups. **(D)** Immunohistochemistry of PFDN2, MYBL2, cyclin D1, cyclin E1 and CDK2 in tumor xenografts.

We used xenograft models to determine the effect of PFDN2 on tumorigenesis *in vivo*. PFDN2-KD/NC HGC27 cells were subcutaneously implanted into nude mice (**Figure 7A**). As shown in **Figures 7B, C**, tumor volume and weight were also significantly decreased in the PFDN2-KD group. Furthermore, IHC analyse of xenograft tissues demonstrated that MYBL2, cyclin D1, cyclin E1 and CDK2 expressions were significantly decreased after PFDN2 knockdown *in vivo* (**Figure 7D**). Taken together, these results demonstrated that PFDN2 could enhance cell cycle progression by promoting MYBL2 expression.

## Discussion

With the rapid development of sequencing technology and bioinformatics, many key genes involved in the development and progression of GC have been identified, which may be effective as tumor biomarkers or therapeutic targets. Previously, we identified PFDN2 as a novel key gene in gastric cancer based on its differential expression between cancer and normal tissues (3). Consistent with our study, other studies have reported that PFDN2 serves as an adverse prognosis marker in gastric cancer, but the function and mechanism of PFDN2 in GC progression remain unknown. In this study, we demonstrated that PFDN2 was highly expressed in GC and that upregulation of PFDN2 was associated with the progression of GC. We further found that PFDN2 could promote cell cycle progression by promoting MYBL2 expression. Mechanistically, we demonstrated that PFDN2 could upregulate MYBL2 expression by facilitating the nuclear translocation of hnRNPD, and thus promoting MYBL2 transcriptional program.

Recently, the PFDN family has gained increasing attention for its aberrant expression in cancers and its potential implications in tumor biology (5). For instance, dysregulated PDFN1 expression has been reported to occur in a variety of cancers, including gastric cancer, lung cancer, and colorectal cancer (6, 17–19); increased expression of PFDN2 has been found in bladder cancer (7); and it has been reported that PFDN4 is highly expressed in breast cancer (20). High PFDN expression could play a potential role in cancer via various mechanisms, including activating the epithelial-mesenchymal transition (19), maintaining cytoskeletal proteins (17) and promoting cancer cell cycle progression (6). We demonstrate here for the first time that PFDN2 could promote gastric cancer cell cycle progression, which extends the understanding of the role of the PFDN family in cancer progression.

Continued cell cycle progression, resulting in sustained proliferation, is one of the hallmarks of cancer (21). The cancer cell cycle is mainly controlled by proteins involved in cell cycle-control signaling pathways. For example, lncRNA SUNO1 promotes cell cycle progression by controlling the YAP1/Hippo signaling pathway (22); miR-218 suppresses gastric cancer cell cycle progression via the CDK6/Cyclin D1/E2F1 axis (23). Among them, the activity of cyclin-dependent kinase (CDK) is thought to be the main driver of cancer cell cycle progression (24). Cell cycle-regulated transcription depends on CDK activity for activation (25). MYBL2, a nuclear transcription regulator, has been shown to have a critical role in regulating CDK expression and activity (26, 27). However, few studies have investigated the role of MYBL2 in GC, especially the mechanisms of MYBL2 upregulation. In our study, we found that MYBL2 could be positively regulated by PDFN2, thus promoting gastric cancer cell cycle progression.

However, our study has some shortcomings. First, we studied the role of PFDN2 only in GC, but the oncogenic role of PFDN2 in other cancers remains unknown. Second, we demonstrated that PFDN2 could promote cancer cell cycle progression and act as an oncogene in GC, but our study could not exclude the possibility that PFDN2 contributes to cancer progression through additional mechanisms. Finally, further studies are required to investigate the detailed mechanism by which the nuclear translocation of hnRNPD regulates MYBL2 expression.

**Figure 8:**
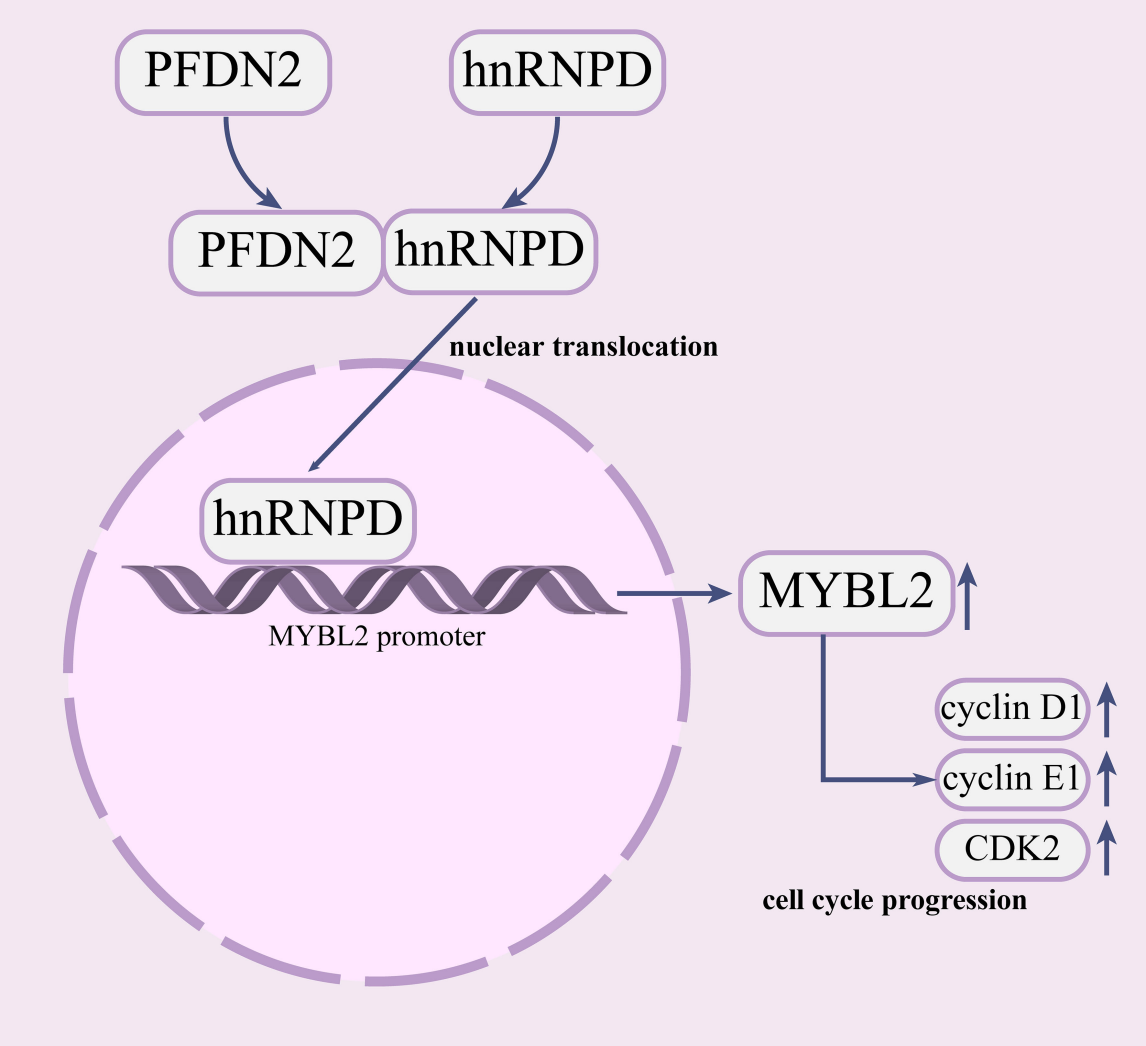
PFDN2 promotes cell cycle progression via the hnRNPD-MYBL2 axis in gastric cancer.

In summary, our study demonstrated that PFDN2 is highly expressed in GC and that upregulation of PFDN2 is associated with the progression of GC. We further found that PFDN2 could promote cell cycle progression by promoting MYBL2 expression. Mechanistically, we demonstrated that PFDN2 upregulates MYBL2 expression by facilitating the nuclear translocation of hnRNPD, and thus promoting MYBL2 transcriptional program. Our study revealed the role of PFDN2 in GC cell cycle progression and may provide a novel tumor marker and a potential target for GC (**Figure 8**).

## Data availability statement

The datasets presented in this study can be found in online repositories. The names of the repository/repositories and accession number(s) can be found in the article/**Supplementary Material**.

## Ethics statement

The studies involving human participants were reviewed and approved by Zhongnan Hospital of Wuhan University ethics committee. The patients/participants provided their written informed consent to participate in this study. The animal study was reviewed and approved by Zhongnan Hospital of Wuhan University ethics committee.

## Author contributions

All authors contributed to the study conception and design. Material preparation, data collection, and analysis were performed by QH, BX, ZD, and TC. The first draft of the manuscript was written by QH and all authors commented on previous versions of the manuscript. HW made significant contributions to the completion of most experiments with the assistance of QH. Additionally, HW provided valuable feedback on the revision of the article and completed a considerable portion of the editing work. All authors contributed to the article and approved the submitted version.
